# The Challenge Arising from New Knowledge about Immune and Inflammatory Skin Diseases: Where We Are Today and Where We Are Going

**DOI:** 10.3390/biomedicines10050950

**Published:** 2022-04-20

**Authors:** Anna Campanati, Emanuela Martina, Annamaria Offidani

**Affiliations:** Department of Clinical and Molecular Sciences, Polytechnic Marche University, 60020 Ancona, Italy; anna.campanati@gmail.com (A.C.); a.ofidani@ospedaliriuniti.narche.it (A.O.)

Skin is the widest and most accessible organ of the human body, and among its functions, the immunological one has been one of the most intriguing and investigated during the last 10 years; so, inflammatory and immune-mediated skin diseases (s-IMID) are considered as useful models to understand which physiopathological pathways are implicated in Th1, Th2, Th17, and Th22 inflammatory diseases.

Basic research has increasingly clarified the complexity of the immunological mechanisms that guide the manifestations of inflammatory skin diseases including psoriasis (PsO), hidradenitis suppurativa (HS), vulvar lichen sclerosus (VLS), chronic lupus erythematosus (CLE), atopic dermatitis (AD), chronic spontaneous urticaria (CSU), and cutaneous systemic sclerosis (SSc).

Advances in the knowledge of the molecular mechanisms and immune biology of inflammatory conditions have not only identified the pathways and cytokines involved, but also verified the contribution made by inflammatory pathways in the various modes of presentation of these diseases. The fundamental merit of these acquisitions is that of having been the prerequisite for identifying the molecular targets for the development of immunotherapies, including biological agents that target specific cell surfaces or extracellular molecules. Additionally, small molecule inhibitors may interfere with intracellular signaling by targeting receptor-associated kinases.

In AD, Park et al. [1], starting from the evidence that alpha-galactosylceramide (a-GalCer), a glycolipid antigen derived from the marine sponge Agelas mauritianus, can selectively activate iNKT cells in a CD1d-dependent manner, investigated whether repeated a-GalCer administration affects the pathogenesis of AD in Va14Tg NC mice. Furthermore, and they examined the effects of this repeated iNKT cell activation on CD4+ T cell polarization during AD development. The authors concluded that long-term exposure to glycolipids such as a-GalCer is associated with an increased incidence of AD. Moreover, the expansion of IL4-producing iNKT cells contributes to an increased Th2-type immune response in a-GalCer-injected Va14Tg NC mice, resulting in a Th1/Th2 imbalance under conventional housing conditions. Therefore, long-term glycolipid administration regimens for immunotherapeutic purposes should consider phenotypic alterations in iNKT cells that may cause undesired side effects.

Di Filippo et al. [2] addressed the more recent scientific acquisitions on CSU, which is a complex disease, with several pathogenetic mechanisms and trigger factors underlying clinical manifestations on the skin [3,4,5]. In CSU, several biologic drugs have been designed to interfere with the underlying inflammatory pathway. Although many biologic drugs are under investigation, omalizumab is currently the only monoclonal antibody approved in patients with severe and treatment-refractory CSU, and this is also the case in children. Although its efficacy and safety profile has been widely demonstrated, high costs represent a barrier to their use, and optimal duration of treatment is yet to be defined. Ligelizumab showed higher affinity compared to omalizumab; therefore, it seems to be a promising alternative, but its efficacy and safety have yet to be evaluated, especially in children.

Contrastingly, in systemic sclerosis, according to Benfaremo et al. [6], although substantial progress has been made in the management of SSc in recent years, disease-modifying therapies are still lacking. Several molecular pathways involved in SSc pathogenesis are currently under evaluation as possible therapeutic targets in clinical trials. These include drugs targeting fibrotic and metabolic pathways (e.g., TGF-, autotaxin/LPA, melanocortin, and mTOR), as well as molecules and cells involved in the persistent activation of the immune system (e.g., IL4/IL13, IL23, JAK/STAT, B cells, and plasma cells).

Similarly, in VLS, despite the advances in the knowledge of the pathogenetic mechanisms of VLS, many aspects and mediators remain unclear. Further investigation is needed to better define the exact sequence of events underlying VLS pathogenesis, key mediators involved in VLS immune response and those which, more than others, trigger an abnormal fibroblast and collagen metabolism, and finally, to what extent keratinocytes and fibroblasts actively participate in VLS pathogenesis; thus, Corazza et al. [7] call for advancement in pathogenetic knowledge to make clear which key mediators are crucial for VLS development. This is the requirement for the identification of extremely selective drugs, such as biologics, capable of modulating or suppressing specific pathogenetic sequences.

These highly selective molecules have changed the natural course of many skin diseases that were not optimally managed until recently as psoriasis, atopic dermatitis, scleroderma, or suppurative hidradenitis.

According to Campanati et al. [8], from the current scientific evidence it emerges that PsO is characterized by the presence of main molecular axes, namely IL-23/IL-17 and TNF-alpha, which can be considered the pathogenic drivers of disease both at a cutaneous and systemic level, and they become revolutionary therapy targets [9]. However, there are many other molecules that “revolve around” these axes, such as IL-22; moreover, both IL-23/IL-17 and TNF-alpha axes are expressed differently among the various organs affected or type of psoriasis expressed [10]. Moreover, the matter is more complex, since psoriasis is a very complex disease whose clinical features range from very mild skin disease to a systemic form with involvement of organs other than the skin [11].

In this sense, Genovese G et al. [12] stated that recent progress in the identification of genetic mutations and immunological mechanisms has promoted a better understanding of pustular PsO pathogenesis and might have important consequences on diagnostic refinement and treatment. They focus also on the promising role of IL36 inhibitors in therapeutic management of patients suffering from pustular psoriasis.

According to Rosi E et al. [13], the current understanding of HS pathogenesis places inflammation as the key actor (the primum movens) in the disease pathogenetic process. Nevertheless, the interplay among genetics, lifestyle, hormonal status, microbiome, and innate and adaptive immune system remains unclear. Besides the role of biologics in actual management of HS, authors report the potential role of artificial intelligence might play a role in HS, firstly in clinical trials, and then becoming useful (for clinicians and patients) in daily clinical practice [14,15].

For other inflammatory skin diseases such as CLE, the understanding of the pathophysiological mechanisms has made it possible to deeply understand the mechanism of action of some commonly used drugs, such as thalidomide. Domingo S. et al. [16] demonstrated that thalidomide’s immunomodulatory anti-inflammatory effect in CLE comprises several mechanisms that include a reduction in predominantly CD8+T cells, and a switch from Th1 to Th2 response. Furthermore, thalidomide reduced NF-kB-related inflammatory cytokines and chemokines via the modulation of IRF4- and AMPK/mTOR-signaling pathways. Following these considerations, authors interestingly concluded that targeting the function of these key molecules may be an alternative to thalidomide for the treatment of CLE.

The extraordinary path of innovation in the therapeutic management of inflammatory and immune-mediated diseases of the skin is also accompanied by the emergence of problems connected with the use of new drugs which, on the one hand, involve extraordinary long-term disease control, unthinkable 20 years ago; they were also accompanied by the emergence of new problems.

In recent years, paradoxical adverse events have been described during treatment of skin inflammatory and immune-mediated skin disease with biologics, particularly with adalimumab [17]. Although, the results of the ADA data can be influenced by the high number of patients who had undergone therapy with ADA, which is the only biological drug currently available for HS; Ruggiero et al. [18] suggests to dermatologists to be aware that faster recognition of these paradoxical reactions by dermatologists would allow for faster correct treatment and better clinical outcomes.

Another dominant issue in the use of biologics in inflammatory and immune-mediated diseases is inherent to the infectious risk. According to the literature, the risk of infection is higher in psoriatic subjects than in the general population [19,20,21,22]. Motolese et al. [23] report that the advent of biotechnological agents on the therapeutic arsenal available for the treatment of moderate-to-severe patients, given the fact that the severity of the disease is a predictor of the level of infectious risk, has raised the question of whether these ‘new’ drugs could be considered a safer option and how they can be used in selected cases. According to the latest literature data and registers, using novel therapies, particularly anti-ILs and anti-PDE4, seem not to have a significant impact on the vulnerability of these patients to infections, thus representing a reassuring option in the management of the disease.

Even the hypothetical neoplastic risk potentially connected with the use of molecular target therapies has long been burdened by speculative evaluation and has represented the prerequisite for the construction of registers and real-life data in the long term, in patients suffering from diseases such as chronic inflammation of the skin undergoing molecular target therapy. In this regard, Li Pomi et al. [24] showed that HS could be associated with an overall risk of cancer and numerous specific cancers such as nonmelanoma skin cancer (NMSC), hematologic malignancies, and metastatic cancer. Among NMSC, squamous cell carcinoma (SCC) can be considered the most common complication arising in long-standing HS. Based on their review, the authors suggest that cautious surveillance and active intervention may be warranted in patients with HS. Moreover, an age-appropriate cancer screening should be offered to all patients, especially those who developed HS later in their life or in long-standing moderate to severe HS with multiple comorbidities.

Finally, the appearance of paradoxical skin reactions in patients with inflammatory skin diseases and undergoing molecular-targeted therapies, which can potentially complicate the patient’s therapeutic process, represents an emerging clinical challenge. Megna et al. [25] reported that data on new-onset of psoriatic arthritis (PsA) in patients with psoriasis treated with biologic drugs are very limited, showing variable and conflicting results. Although treating psoriasis with biologic drugs reduces the risk of PsA development [26,27,28], clinicians must also keep in mind the risk of new-onset PsA in patients undergoing biologic treatment, so PsA screening should be strongly recommended for each follow-up visit. Authors concluded that further studies are needed to clarify the pathogenesis of PsA and eventual risk factors and deepen the correlation between biologic therapy and new-onset PsA to allow finding predictive factors that could help in preventing these events and choosing the best tailored-tail therapy for each patient.

In conclusion, inflammatory and immune-mediated diseases of the skin (s-IMID) represent a spectrum of diseases, which are highly variable in terms of clinical expression, prognosis, complications, and response to therapy. Furthermore, within each single category of disease attributable to s-IMID, each patient can be considered a “per-se” expression of disease, for disease phenotype, site of involvement, related symptoms, comorbidities, and ability to respond/tolerate to current treatments. All data and considerations included into this review reflect the state of the art in this clinical and research field. Many of the current reported considerations offer significant cultural contributions, moving clinicians toward the “patient-tailored” treatment, according to the “precision medicine model”. In the future, investigations on genetic polymorphism, microRNAs, serum biomarkers, and combined data analysis with artificial intelligence may guide future studies to identify different clinical profiles for every type of s-IMID, with the purpose of further improving knowledge on disease development and optimizing the target approach to the patients.

## References

[1] Park H.J., Kim T.-C., Park Y.H., Lee S.W., Jeon J., Park S.-H., van Kaer L., Hong S. (2021). Repeated α-GalCer Administration Induces a Type 2 Cytokine-Biased iNKT Cell Response and Exacerbates Atopic Skin Inflammation in Vα14Tg NC/Nga Mice. Biomedicines.

[2] Di Filippo P., Russo D., Attanasi M., Di Pillo S., Chiarelli F. (2021). Immunological Targets of Biologic Drugs in Allergic Skin Diseases in Children. Biomedicines.

[3] Campanati A., Gesuita R., Giannoni M., Piraccini F., Sandroni L., Martina E., Conocchiari L., Bendia E., Di Sario A., Offidani A. (2013). Role of small intestinal bacterial overgrowth and Helicobacter pylori infection in chronic spontaneous urticaria: A prospective analysis. Acta Derm. Venereol..

[4] Damiani G., Diani M., Conic R.R.Z., Colli L., Ferrucci S., Martina E., Offidani A.M., Pigatto P.D.M. (2019). Omalizumab in Chronic Urticaria: An Italian Survey. Int. Arch. Allergy Immunol..

[5] Martina E., Damiani G., Grieco T., Foti C., Pigatto P.D.M., Offidani A. (2021). It is never too late to treat chronic spontaneous urticaria with omalizumab: Real-life data from a multicenter observational study focusing on elderly patients. Dermatol. Ther..

[6] Benfaremo D., Svegliati S., Paolini C., Agarbati S., Moroncini G. (2022). Systemic Sclerosis: From Pathophysiology to Novel Therapeutic Approaches. Biomedicines.

[7] Corazza M., Schettini N., Zedde P., Borghi A. (2021). Vulvar Lichen Sclerosus from Pathophysiology to Therapeutic Approaches: Evidence and Prospects. Biomedicines.

[8] Campanati A., Marani A., Martina E., Diotallevi F., Radi G., Offidani A. (2021). Psoriasis as an Immune-Mediated and Inflammatory Systemic Disease: From Pathophysiology to Novel Therapeutic Approaches. Biomedicines.

[9] Campanati A., Ganzetti G., Giuliodori K., Molinelli E., Offidani A. (2016). Biologic Therapy in Psoriasis: Safety Profile. Curr. Drug Saf..

[10] Radi G., Campanati A., Diotallevi F., Bianchelli T., Offidani A. (2021). Novel Therapeutic Approaches and Targets for Treatment of Psoriasis. Curr. Pharm. Biotechnol..

[11] Ganzetti G., Campanati A., Santarelli A., Pozzi V., Molinelli E., Minnetti I., Brisigotti V., Procaccini M., Emanuelli M., Offidani A. (2015). Involvement of the oral cavity in psoriasis: Results of a clinical study. Br. J. Dermatol..

[12] Genovese G., Moltrasio C., Cassano N., Maronese C.A., Vena G.A., Marzano A.V. (2021). Pustular Psoriasis: From Pathophysiology to Treatment. Biomedicines.

[13] Rosi E., Fastame M.T., Scandagli I., Di Cesare A., Ricceri F., Pimpinelli N., Prignano F. (2021). Insights into the Pathogenesis of HS and Therapeutical Approaches. Biomedicines.

[14] Walss M., Anzengruber F., Arafa A., Djamei V., Navarini A.A. (2021). Implementing Medical Chatbots: An Application on Hidradenitis Suppurativa. Dermatology.

[15] Gomolin A., Netchiporouk E., Gniadecki R., Litvinov I.V. (2020). Artificial Intelligence Applications in Dermatology: Where Do We Stand?. Front. Med..

[16] Domingo S., Solé C., Moliné T., Ferrer B., Cortés-Hernández J. (2021). Thalidomide Exerts Anti-Inflammatory Effects in Cutaneous Lupus by Inhibiting the IRF4/NF-κB and AMPK1/mTOR Pathways. Biomedicines.

[17] Campanati A., Moroncini G., Ganzetti G., Pozniak K., Goteri G., Giuliano A., Martina E., Liberati G.L., Ricotti F., Gabrielli A. (2013). Adalimumab Modulates Angiogenesis in Psoriatic Skin. Eur. J. Inflamm..

[18] Ruggiero A., Martora F., Picone V., Marano L., Fabbrocini G., Marasca C. (2022). Paradoxical Hidradenitis Suppurativa during Biologic Therapy, an Emerging Challenge: A Systematic Review. Biomedicines.

[19] Amerio P., Amoruso G., Bardazzi F., Campanati A., Cassano N., Conti A., Gisondi P., Guarneri C., Mazzotta A., Piaserico S. (2013). Detection and management of latent tuberculosis infections before biologic therapy for psoriasis. J. Dermatol. Treat..

[20] Bardazzi F., Magnano M., Campanati A., Loconsole F., Carpentieri A., Potenza C., Bernardini N., Di Lernia V., Carrera C., Raone B. (2017). Biologic Therapies in HIV-infected Patients with Psoriasis: An Italian Experience. Acta Derm. Venereol..

[21] Gisondi P., Cazzaniga S., Chimenti S., Maccarone M., Picardo M., Girolomoni G., Naldi L., Griseta V., Miracapillo A., Psocare Study Group (2015). Latent tuberculosis infection in patients with chronic plaque psoriasis: Evidence from the Italian Psocare Registry. Br. J. Dermatol..

[22] Campanati A., Ganzetti G., Martina E., Giannoni M., Gesuita R., Bendia E., Giuliodori K., Sandroni L., Offidani A. (2015). *Helicobacter pylori* infection in psoriasis: Results of a clinical study and review of the literature. Int. J. Dermatol..

[23] Motolese A., Ceccarelli M., Macca L., Pomi F.L., Ingrasciotta Y., Nunnari G., Guarneri C. (2022). Novel Therapeutic Approaches to Psoriasis and Risk of Infectious Disease. Biomedicines.

[24] Li Pomi F., Macca L., Motolese A., Ingrasciotta Y., Berretta M., Guarneri C. (2021). Neoplastic Implications in Patients Suffering from Hidradenitis Suppurativa under Systemic Treatments. Biomedicines.

[25] Megna M., Ocampo-Garza S.S., Potestio L., Fontanella G., Gallo L., Cacciapuoti S., Ruggiero A., Fabbrocini G. (2021). New-Onset Psoriatic Arthritis under Biologics in Psoriasis Patients: An Increasing Challenge?. Biomedicines.

[26] Gisondi P., Bellinato F., Targher G., Idolazzi L., Girolomoni G. (2022). Biological disease-modifying antirheumatic drugs may mitigate the risk of psoriatic arthritis in patients with chronic plaque psoriasis. Ann. Rheum. Dis..

[27] Felquer M.L.A., LoGiudice L., Galimberti M.L., Rosa J., Mazzuoccolo L., Soriano E.R. (2022). Treating the skin with biologics in patients with psoriasis decreases the incidence of psoriatic arthritis. Ann. Rheum. Dis..

[28] Salaffi F., Di Carlo M., Luchetti M.M., Di Donato E., Campanati A., Benfaremo D., Nicolini M., Carotti M., Giacchetti A., Ganzetti G. (2018). A validation study of the Simple Psoriatic Arthritis Screening (SiPAS) questionnaire to screen psoriasis patients for psoriatic arthritis. Clin. Exp. Rheumatol..

